# Thresholds in the Species–Area–Habitat Model: Evidence from the Bryophytes on Continental Islands

**DOI:** 10.3390/plants12040837

**Published:** 2023-02-13

**Authors:** Guangyu Luo, Ruoling Huang, Shuiliang Guo, Dandan Li, Jun Yang, Feng Zhang, Jing Yu

**Affiliations:** College of Life Sciences, Shanghai Normal University, Shanghai 200234, China

**Keywords:** habitat, threshold, small-*choros* effect, species richness, the *choros* model

## Abstract

Aim: To clarify whether (1) there are thresholds in the species–area–habitat relationship for bryophytes and potential mechanisms, (2) such thresholds vary among different bryophyte groups, and (3) *choros* is better than area or habitat alone in the prediction of SR. Location: Islands in central and southern Zhejiang, China. Methods: We investigated the species richness (SR) of five bryophyte groups (total bryophytes, total mosses, liverworts, acrocarpous mosses, and pleurocarpous mosses) and habitat types on 66 islands. By using four threshold models, the logarithmic and the power models, we quantified their SR–*choros* relationships (SKRs), species–area relationships (SARs), and species–habitat relationships (SHRs). We also conducted path analyses to detect the direct effects of area per se and habitat per se on the SR. Results: The AICc values of the SKR models were overall smaller than those of the respective SAR and SHR models. The left-horizontal two-threshold model was best for the SKRs. A phenomenon (the small-*choros* effect, SCE) in which SR independently varied choros below a given threshold was detected. The SCE thresholds were smaller in mosses than in liverworts and in acrocarpous mosses than in pleurocarpous mosses. No direct and positive effects of habitat per se on the SR were detected below *choros* thresholds for all five groups. Main conclusions: There were two thresholds and SCEs in the SKRs of all five bryophyte groups. The SCEs likely resulted from the elimination of the direct and positive effects of habitat diversity on the SR of the bryophytes on small *choros* islands. The SCE thresholds were high for species groups sensitive to environments. *Choros* was better than area or habitat alone in determining the SR of the bryophytes on continental islands.

## 1. Introduction

An essential goal of ecology and biogeography is to identify the factors determining species richness (SR). The area has been considered one of the most important determinants of SR [1]. There are many hypotheses that have been proposed to explain the relationships between SR and area (SARs), but the two most important are the habitat diversity hypothesis [2] and the area per se hypothesis [3,4,5,6,7,8]. Although ecologists attempted to establish the primacy of one over the other, area and habitat were strongly correlated, and both influenced SR [1,6,9]. Triantis et al. thought that SR does not depend solely on area or habitats but on both of them, and they introduced the term “*choros*” as the combined effect of habitat diversity (*H*) and area (*A*) on SR [10]. The *choros* (*K*) of a given region is the multiplication of the area (*A*) and the number of habitat types (*H*) of the region (*K* = *A × H*). The relationship of *SR* with *K* (SKR) could be quantified by the *choros* power model, *S* = *c × K^z^*.

The *choros* model has been successfully applied to account for SR for many biotas in different ecosystems and proved to be better than the classic SAR power model [11,12,13,14,15,16,17,18,19]. However, *choros* was not always better than area or habitat alone in the prediction of SR [10,20]. Regarding the applicability of the *choros* model in plants, all studies were conducted on vascular plants; we do not know the performance of the model in other special plant taxa, such as bryophytes.

Bryophytes are unique among green land plants. They have unique diversity and distribution patterns because of their special physiological and ecological features. Contrary to vascular plants, bryophytes developed a poikilohydric strategy, which allows them to absorb water over their whole surface by capillarity [21]. Bryophytes are rather sensitive to habitat changes [22,23]. Furthermore, bryophytes often have long-distance dispersal abilities, and many have wide distribution ranges [24,25]. Additionally, bryophytes have rapid population colonization and extinction rates [26]. Therefore, bryophytes represent an alternative strategy for survival in terrestrial environments, and the ecological patterns and mechanisms of bryophytes likely differ from those of vascular plants [27]. We speculated that the relationships between SR and *choros* of bryophytes likely differ from those of other land green plants, but we know nothing about this.

Ecologically, a threshold is considered a point of abrupt change of the response variable of an ecological process against a measure in environmental factor [28]. In many studies of ecological thresholds, response variables often were the indices of ecological health, and the environmental factors were human disturbances [29]. Ecological thresholds are important for understanding the requirements of biotas and evaluating to what extent the environmental changes will have critical consequences on ecological processes, thus providing objective conservation strategies [28].

Thresholds have been detected in SARs, species richness–habitat relationships (SHRs), and phylogenetic diversity–area relationships (PDARs) in many biotas across different ecosystems [28,30,31,32,33,34,35,36,37]. By detecting the thresholds in SARs, ecologists found the small-island effect (SIE) [31,33,37,38,39,40,41]. The SIE is a phenomenon where different SARs exist on smaller islands compared with larger islands. More exactly, SR in smaller islands (below a threshold) independently varies from island size (the SIE sensu stricto) or increases at a lesser rate than that in larger islands (above the threshold; the SIE sensu lato) [35,42,43,44]. Because thresholds often exist in SARs and SHRs, while area and habitat are strongly interconnected [6], and *choros* is the combination of area and habitat [10], we speculated that there likely also exist thresholds in SKRs. If there are thresholds in SKRs, then we would expect a phenomenon in which SR independently varies of *choros* on the islands below a certain threshold or increases at a lesser rate than that on the islands above the threshold. Here we refer to this potential phenomenon as the “small-*choros* effect (SCE)”, which is analogous to the SIE. However, we need evidence to confirm the existence of such thresholds and the SCEs in SKRs.

Bryophytes are an informal group mainly consisting of liverworts and mosses [45]; the latter was essentially divided into acrocarpous and pleurocarpous mosses [46,47]. There are differences in environmental adaptation between liverworts and mosses and between acrocarpous and pleurocarpous mosses [44,45]. Mosses are generally more drought tolerant than liverworts [48,49]. Many liverworts prefer shady habitats [50]. Generally speaking, acrocarpous mosses, often in turfs and cushions, usually dominate in sunny, dry, and xeric habitats, whereas pleurocarpous mosses, often in mats, wefts, tail, and fan forms, frequently dominate in shady, humid, and mesic to hydric sites [44]. If there are thresholds in SKRs, we speculated that the thresholds would vary among different bryophyte groups. Therefore, we needed to conduct studies to know the difference in thresholds among different bryophyte groups.

The central and southern Zhejiang province of China comprises ca. 1200 islands [51]. These continental islands have typical fragmented landscapes, varying in their area and habitat types. Thus, they are an ideal ecosystem for us to investigate the thresholds and SCEs in SKRs of the bryophytes in fragmented habitat systems. Our aims are to clarify (1) the applicability of the *choros* power model in predicting the SR, (2) thresholds and SCEs in SKRs, and (3) the differences in the choro thresholds of the SKRs among different groups of bryophytes on continental islands.

## 2. Results

The accumulative species increased with accumulative specimens, well following the asymptotic model. The errors between the collected species number and the expected maximum species number were all less than 6.58% except for the Chaoyanhoushan Island (10.13%) (Table S3), indicating that sampling on larger islands was adequate.

A total of 236 bryophyte species and 25 habitat types were recorded on the 66 islands (Tables S4 and S5). Among these species, 30 were liverworts, and 206 were mosses. Acrocarpous mosses (116 species, 49.15%) were richer than pleurocarpous mosses (85, 36.02%) (Table S6).

Among the six SKR models that we tested, in the prediction of the SR of the five bryophyte groups in the study region, the left-horizontal two-threshold models (ΔAICc 0–3.50, mean 1.02) were overall the best, followed by the power SKR model (0–9.35, 2.05), the two-threshold models (0–5.95, 2.58), the one-threshold models (4.12–8.59, 6.40), the left-horizontal one-threshold models (3.33–14.75, 8.72), and the simple logarithmic model (18.03–44.03, 30.36) (Table 1). The two-threshold models were better than the one-threshold models in predicting the SR.

**Figure 1:**
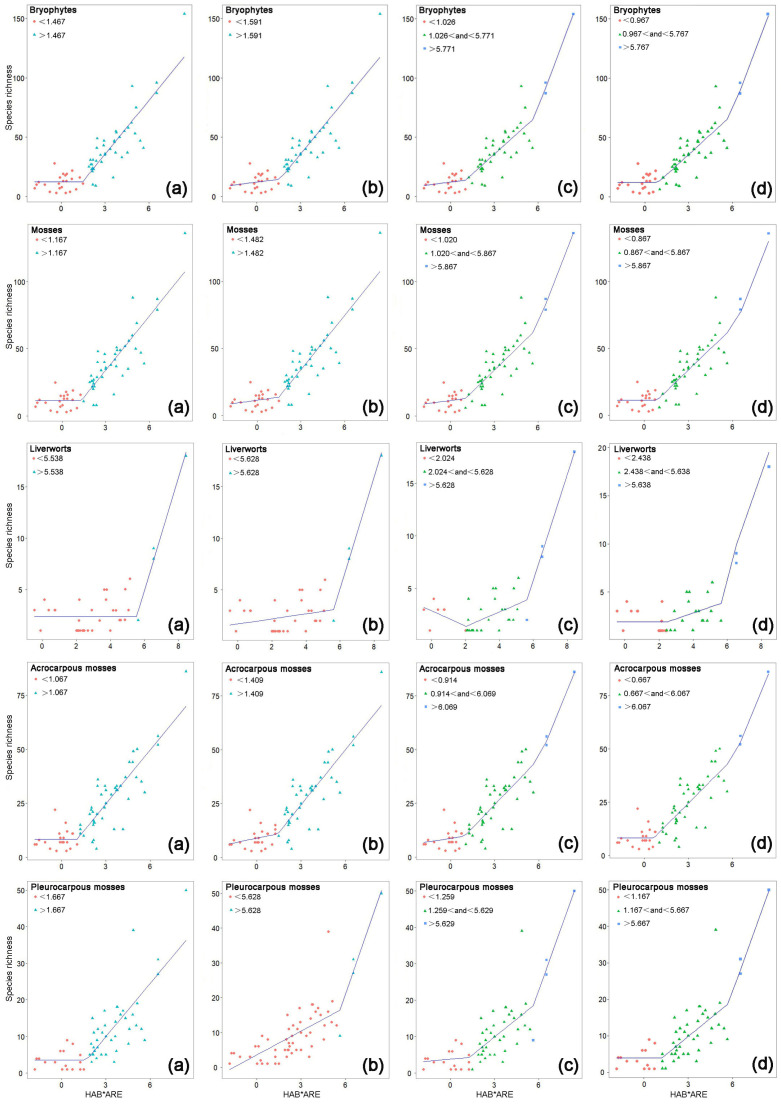
Six SKR models for five groups of bryophytes on 66 islands. Note: (**a**–**d**) represent the left-horizontal one-threshold model, one-threshold model, two-threshold model, and left-horizontal two threshold-model, respectively. HAB * ARE represents ln (choros) (ln (number of habitat types * area km^2^)).

The SCEs sensu stricto were identified by using the left-horizontal two-threshold SKR models. The first threshold of the two-threshold model represents the upper limit of the SCE. The *choros* threshold (nh∙km^2^) of the SCEs sensu stricto was 2.63 for total bryophytes, larger in liverworts (11.45) than in total mosses (2.38) and larger in pleurocarpous mosses (3.21) than in acrocarpous mosses (1.95) (Table 1, Figure 1).

Second *choros* thresholds (nh∙km^2^) were detected by using the left-horizontal two-threshold SKR models for total bryophytes (319.578), total mosses (353.188), acrocarpous mosses (431.385), pleurocarpous mosses (289.166), and liverworts (280.9) (Table 1). Above the second threshold, the SR steeply increased with increasing *choros*. Among 236 species on the 66 islands, 43 were recorded only from the three largest islands above the second threshold (319.58 nh∙km^2^).

According to the thresholds of the left-horizontal two-threshold SKRs, 66 islands were divided into small and large *choros* island groups. The deviation values in the small *choros* island group (below a certain *choros* threshold) were significantly larger than those in the island group (above the threshold) for all five bryophyte groups (Table S7). The results based on null model analyses further confirmed the SCEs for the bryophytes on continental islands.

The above results provided strong evidence to support the existence of thresholds and SCEs in the SKRs of all five groups of the bryophytes on these 66 continental islands.

Based on the ∆AICc values, among the SAR and SHR models we tested, the left-horizontal two-threshold models are essentially the best for the SARs of all five groups and for the SHRs of all five groups, except the liverworts. The left-horizontal one-threshold SHR model was best for the liverworts. Therefore, thresholds also existed in the SARs and SHRs of all five groups of the bryophytes in the study region (Tables S8 and S9 and Figures S1 and S2). Two-threshold models could overall be applied to the SARs and SHRs of the bryophytes in the study region.

Almost all of the AICc values of the six SKR models were smaller than those of the corresponding SAR models for the five groups. The adjusted r^2^ values of the SKR models were essentially larger than those of the SAR models (Table 2). Our results indicated that *choros* was better than the area in predicting the SR of all five bryophyte groups in the study region (Table 2 and Table 3).

*Choros* was better than, or at least similar to, habitat number in predicting the SR of all five groups except pleurocarpous mosses. For pleurocarpous mosses, *choros* was also better than habitat number in predicting its SR in the logarithmic and power models but was not in the threshold models (Tables S9–S11).

According to the path analysis we conducted, the direct effects of habitat number per se on the SR of total bryophytes, total mosses, and acrocarpous mosses were eliminated on the islands below a *choros* threshold of 1.13 nh∙km^2^. This threshold corresponds to that of the 13th island among the 66 islands arranged in the order of *choros* from smallest to largest (Figure S3 and Table S12). Similarly, the *choros* thresholds (nh∙km^2^) for the pleurocarpous mosses and the liverworts were 1.44 and 37.57, respectively. Therefore, the SR of bryophytes on the small *choros* islands (below a threshold) independently varied in habitat number per se.

However, there were direct and positive effects of area per se on the SR of all five bryophyte groups across all the islands where they inhabited. Namely, even in the small choros islands area per se still essentially exerted direct and positive effects on the SR of the bryophytes (Figure S4).

The above results showed that, on large choros islands above a certain choros threshold, both habitat number per se and area per se exerted positive effects on the SR of the bryophytes, but on small choros islands below the threshold, the direct and positive effects of habitat per se on the SR were eliminated.

## 3. Discussion

### 3.1. The Creditability of the SCEs and Thresholds

In some previous studies, there were possible sampling biases for large islands because the number of sampling points per unit area sometimes declined with the increase in island size [52]. In the present study, the relationship between accumulative species number and accumulative specimens (after randomization) well followed the asymptotic model for the first eight largest islands. The errors between the collected species number and the expected maximum species number were all less than 6.58%, except for the Chaoyanhoushan Island (10.13%) (Table S3). Therefore, our sampling efforts were overall adequate and acceptable.

The thresholds and the SCEs in the SKRs of the bryophytes in the study region were convincing. Firstly, we fitted four continuous piecewise models and selected the best models by comparing their ∆AICc values in the same S-space with those of the simple logarithmic model and the Arrhenius’ SAR power model [31,36,38,39]. Secondly, we used a null model to further confirm the existence of SCEs in the SKRs for all five bryophyte groups [42].

### 3.2. Thresholds in SKRs and Variations among Different Bryophyte Groups

Most piecewise SARs consist of only one threshold of the SIE. Lomolino and Weiser [53] proposed a two-threshold SAR model with an ecological threshold of the SIE for small islands and an evolutionary threshold relevant to a speciation phenomenon in situ for large islands. Two area thresholds and SIEs in SARs have been detected in the bryophytes in the Zhoushan Archipelago, China [54], the reptiles of the Seribut Archipelago and the amphibians in the West Indies [40], and the vascular plants in the Aegean Archipelago, Greece [33]. We detected two thresholds in the SKRs, SARs, and SHRs of the bryophytes on continental islands. Our findings further confirmed that thresholds were not limited to SARs and also existed in the relationships of SR with other environmental factors. Two-threshold ecological processes were not accidental ecological phenomena.

Triantis et al. found that the *choros* model was better than the classic SAR model in predicting the SR of land snails in the Skyros Archipelago in the central Aegean Sea (Greece), but the explanatory power of the *choros* model declined on small islands [18]. Their findings indicated a potential *choros* threshold in their SKR of the land snail. Our results unequivocally confirmed the existence of thresholds in SKRs.

Small continental islands are often characterized by relatively dry and sunny habitats because of the lack of fresh water, sparse vegetation, and salt-exposed habitats [34]. The islands with large *choros* values are very likely large islands. Large islands often possess more habitats with forests and sufficient freshwater than smaller islands. Compared with mosses, liverworts prefer forest and shady habitats [48,49,50]. Staniaszek-Kik et al. thought that liverworts were more likely than mosses to be specialists [55]. Acrocarpous mosses usually prefer sunny, dry, and xeric habitats, whereas pleurocarpous mosses frequently prefer shady, humid, and mesic to hydric habitats [44]. Therefore, large *choros* islands would regularly provide more opportunities than small *choros* islands for the establishment of liverworts (compared with mosses) and pleurocarpous mosses (compared with acrocarpous mosses), and the threshold of the SCEs should be larger in liverworts than in mosses and larger in pleurocarpous mosses than in acrocarpous mosses. The above inference was confirmed by our findings. For example, on the 66 islands, the proportion of liverworts to mosses was higher on large *choros* islands above the second threshold (0.151) than on the small *choros* islands below the first threshold (0.138), and the proportion of pleurocarpous mosses to acrocarpous mosses was also higher on the large islands (0.670) than on the small islands (0.383). Namely, the *choros* threshold of the SCE was likely large for species groups with relatively high habitat specificity and sensitivity. Our results indicated that the *choros* value of a reserve for the conservation of species with high habitat specificity should be comparatively large.

### 3.3. Mechanisms of the Thresholds in SKRs

In the left-horizontal two-threshold SKRs (Figure 1 and Table 1), the first threshold revealed the SCEs in the SKRs for the bryophytes in the study region. The SCEs in the SKRs of the bryophytes in the continental islands are possible due to the following two reasons: Firstly, the islands below the first *choros* threshold are likely smaller islands. On these small islands, extinction rates of bryophyte populations vary independently of *choros* because of episodic catastrophic disturbances such as storms or other stochastic events [56]. Secondly, habitat availability is the main driver of SR [35,38]. This is indeed the case that habitat diversity per se exerts positive effects on the SR of the bryophytes on large choros islands (Figure S3). However, the islands below a given choros threshold will contain limited habitat types, which causes irregular changes in the number of habitat types on these islands. Our path analysis showed that the direct and positive effects of habitat number per se on the SR of the bryophytes were eliminated on the small choros islands (Figure S3), while those of area per se still existed. Therefore, it was habitat per se rather than area per se that caused the SCEs in the SKRs.

Above the second threshold, the SR steeply increased with increasing *choros*. This phenomenon was possibly due to the following two reasons. Firstly, there possibly existed a high immigration rate of the bryophytes because human activities likely increased the dispersal chances of bryophytes from the mainland and adjacent islands. In the study region, the islands above the second threshold were likely very large islands, which have a large number of residents and are not far from the mainland. Bryophytes are able to propagate via various vegetative organs, such as rhizoidal gemmae, axillary gemmae, brood bodies, detaching leaves, stems, buds, and leaf fragments. These organs are easily dispersed by human activities [57], for example, *Marchantia emarginata* Reinw., Blume and Nees, and *M. subintegra* Mitt. Secondly, we could expect lesser habitat fragmentation and disturbance and higher “quality” and stability of ecotopes on large islands, which is positive for the population maintenance of rarer species.

### 3.4. Implications of Choros and the Threshold SKRs

Bryophytes were often considered as having a long-distance dispersal capability by spores, even by vegetative propagules [57,58]. The isolation degree of continental islands is weaker than that of ocean islands. The dispersal capacities of a focal biota and the isolation degree of the ecosystem would influence the parameters and fit-goodness of the power SAR model for biotas on insular ecosystems [6,56]. The *choros* model assumed that the combination of area and habitat was better than the area in predicting SR [10]. However, no work has been conducted to verify the applicability of the *choros* model for bryophytes on continental islands. Our results showed that *choros* was overall better than area per se and habitat per se in predicting the SR of bryophytes, except for the threshold SHR modes for the pleurocarpous mosses.

The *choros* model tends to be more effective as the correlation between area and habitat decreases [10]. For the 58 islands with pleurocarpous mosses in the study region, the correlations between area and habitat number in different island groups (identified by the threshold in their SHRs) were relatively high. This is one possible reason for the unsatisfactory performance of the threshold SKR models compared with the threshold SHR models in predicting the SR of the pleurocarpous mosses.

Triantis et al. thought that *choros* could also be used for detecting SIEs [10]. However, the multiplication of the area with the number of habitat types would magnify the variation in predictor variables, potentially introduce environmental stochastics for the smaller islands, and bias the detection of SIEs. Therefore, it is not very logical to use *choros* to detect SIEs. Here, we introduced a new concept, the small-*choros* effect, in the SKRs.

Chen et al. suggested that habitat diversity should be included in the analyses of SIEs [38]. Triantis et al. considered the *choros* model as a stepping-stone in the understanding of SIEs [10]. However, the former stressed the direct effect of area on SR independent of habitat diversity in their detection of SIEs, and the latter implied that SIEs could be detected by using *choros* as an explanatory variable. By using path analysis with the sequential exclusion of islands from the largest to smallest, Triantis et al. [35], Wang et al. [41], and Chen et al. [38] detected SIEs independently of habitat diversity. According to their viewpoints, the SIE appears when and where area ceases to influence species richness directly. However, area and habitat diversity are tightly interconnected, and they should exert a combined effect on biodiversity [10]. In the practice of nature reserve management, we thought that *choros* was more useful than either area or habitat. For a biota with threshold SKRs in a given reserve, we could expect that only when the *choros* values of the reserve are larger than the threshold, enlarging *choros* (by increasing area or habitat type, or both) could be effective for the conservation of the biota in reserve. The detection of thresholds and SCEs in SKRs was of practical value in the design of reserves, especially for the conservation of bryophytes. It is the total effect of area and habitat that determines the biodiversity of the region. Therefore, biodiversity could be improved by increasing the *choros* value via the construction of diverse habitat types, which was especially important for bryophytes because the habitat types suitable for bryophytes are relatively and easily constructed or formed by human interventions [59,60,61,62,63].

The structure of the *choros* model is analogous to the ‘species-energy’ relationship with a multiplicative formula of the area and actual evapotranspiration (AET) (area × AET) to predict the energetic resources. In the determination of SR, the area and elevation often have a positive interaction effect [64]. We suggest including elevation in the *choros* model to further improve the predictive power of SR.

### 3.5. Some Notes of Caution

The improvement in the fit of the *choros* model comes from the inclusion of habitat information in the model. However, habitat types are difficult to define across different biotas in a standardized way [18]. Therefore, the major obstacle to the application of the *choros* model was the disparity between how organisms experience habitat and how ecologists have operationally defined habitat [18]. Our findings indicated that we could develop more powerful models of SR by quantifying habitat types that are truly mechanistically related to the species’ ecological requirements [65]. Bryophytes are more sensitive to microhabitats and substrates than vascular plants [22,23]. The habitat types of bryophytes are greatly different from those of vascular plants. Among the 25 habitat types in the study region, many were recorded at the microscale, and special for bryophytes (such as a cemetery, ditch and pond, low herbosa, stone step, flower bed and flowerpots, and soil roads under forests). Therefore, the six SKR models were overall better than the SAR models for all five groups of bryophytes in the study region. In the future, in order to further improve the fit of the choros model of bryophytes, we could define habitats of bryophytes by using a habitat matrix with different biotopes (or landscapes) in the vertical axis (such as vegetable fields, coniferous forests, broad-leaved forests, mixed coniferous forests, shrubs, herbosas, mountain streams, and orchards) and elements of the biotopes (microhabitats or substrates) in the horizontal axis (such as stone, fallen wood, rocky outcrop, rocky mound, and various slopes) [18].

Considering the complexity, multifaceted, and difficulties in quantifying habitat diversity or defining habitat types related to a focal biota in an objective and repeatable fashion, is there some index to substitute habitat diversity? Island area and elevation are two important determinants of SR, and their positive interaction effects on SR were also identified [64]. More importantly, habitat diversity often increases with the increase in elevation [66]. Although the correlations between habitat diversity and elevation vary with space scales and the definitions of habitat types, island elevation has been used as a surrogate of habitat diversity [65]. The structure of the *choros* model is not new. In the future, it is possible that we substitute habitat × area with elevation × area to improve the predictive power of SR and to make the prediction repeatable and comparable among different studies.

## 4. Materials and Methods

### 4.1. Study Area

**Figure 2:**
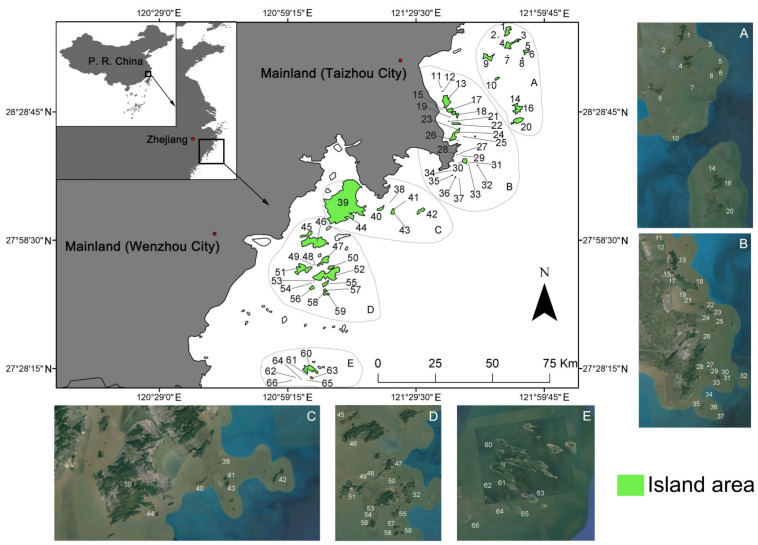
Localities of 66 continental islands in central and southern Zhejiang, China.

A total of 66 continental islands were surveyed (Table S1 and Figure 2). These islands administratively belong to Taizhou City and Wenzhou City in the central and southern Zhejiang province, China (28°47′40.01″–27°25′15.04″ N, 121°0′48.46″–121°55′17.18″ E). They are scattered in a discrete band, lying from the southwest to northeast in the form of archipelagos. Most of these islands are located near the Chinese mainland and have a rocky coastline [67]. The study region covers a land area of 349.47 km^2^, with the largest island being Yuhuandao Island (39: 184.55 km^2^). Twenty-nine islands are larger than 1 km^2^, and 35 have no residents.

The study region belongs to the typical subtropical ocean monsoon zone [51]. The average yearly temperature is ca. 17.3 °C. The average monthly temperature is highest in August (ca. 28.2 °C) and lowest in February (ca. 7.4 °C). The annual frost-free period lasts from 280 days to 334 days. The annual rainfall varies from 1353 mm to 1410.6 mm [68].

### 4.2. Bryophyte Inventory

We surveyed bryophyte flora on the 66 islands from April 2018 to May 2019. We spent a relatively comparable time on each island to collect bryophyte specimens. On small islands (<5.0 km^2^), our surveys essentially covered the entire island. On large islands, we collected specimens in as many landscapes and habitat types as possible via a variety of roads available to us.

The landscapes included hillsides, mountain creeks, rock walls, various forests, bushes, meadows, agricultural lands and plantations, villages and small towns, wastelands, etc. On each island, we continued collecting specimens until no additional species and habitat types were found. The above scheme ensured that we obtained a relatively complete list of species types for each island.

Preliminary identification of family and genus was completed in the field. All specimens were identified by species in the laboratory by using a microscope. Voucher specimens were deposited in the bryophyte herbarium of Shanghai Normal University (SHTU). The nomenclature followed Jia and He [69].

### 4.3. Surveys of Habitat Types

The habitat types on each island were enumerated using the aspects of the habitat types known to be important to bryophyte distribution [70,71,72,73,74,75]. Definitions of each habitat type are listed in Table S2. We recorded habitat types on each island mainly according to our observations in situ at the locations where we collected specimens.

### 4.4. Data Analysis

To detect whether there are thresholds and SCEs in the SKRs and whether *choros* is better than area or habitat richness alone in predicting the SR of the bryophytes in the study region, we used the following four threshold models and two classic models without thresholds (the simple logarithmic model and the power model) [31,34,36,38,39].

Power model: *S* = c × *E* ^^*z*^

Simple logarithmic model: *S* = *C* + *Z*_1_ × *E*

Left-horizontal one-threshold model: *S = C + | Z*_2_ × (*E − T*_1_) *|* (*E > T*_1_)

One-threshold model: *S* = *C* + *Z*_1_ × *E* (*E* ≤ *T*_1_) + | *Z*_1_ × *T*_1_ + *Z*_2_ × (*E* − *T*_1_) | (*E* > *T*_1_)

Left-horizontal two-threshold model: *D* = *C* + | *Z*_2_ × (*E* − *T*_1_) | (*T*_2_ ≥ *E* > *T*_1_) + | *Z*_2_ × (*T*_2_ − *T*_1_) + *Z*_3_ × (*E* − *T*_2_) | (*E* > *T*_2_)

Two-threshold model: *D* = *C* + *Z*_1_ × *E* (*E* ≤ *T*_1_) + | *Z*_1_ × *T*_1_ + *Z*_2_ × (*E* − *T*_1_) | (*T*_2_ ≥ *E* > *T*_1_) + | *Z*_2_ × (*T*_2_ − *T*_1_) + *Z*_3_ × (*E* − *T*_2_) | (*E* > *T*_2_)

*S* is the species number of a given island; *E* is an explanatory variable of the island, which is ln (*choros*, number of habitat types * km^2^) for SKRs, ln (area, km^2^) for SARs, and number of habitat types for SHRs; *C* is an intercept; *T*_1_ and *T*_2_ are the first and second *choros* thresholds, respectively; and *Z*_1_, *Z*_2_, and *Z*_3_ are the slopes of the first, second, and third segments, respectively. The logical expressions in parentheses return a value of 1 if true and 0 if false [42].

Inspired by Wang et al. [36] and Chen et al. [31,38,39], we used the above two classic models without thresholds as references to detect thresholds and SCEs by comparing their Akaike information criterion corrected (AICc) [76].

AIC = 2 × (*p* + 1) + *n* × ln (SSE/*n*) + *n* + *n* × ln (2 × π)

and

AICc = AIC + 2 *p* × (*p* + 1)/(*n* − *p* − 1),

where *n* is the number of islands, SSE is the sum of squared estimates of errors, π = 3.14159, and *p* is the number of parameters.

When calculating AICc, the variance was considered as an additional parameter. Thus, the parameter *p* for the left-horizontal one-threshold model, one-threshold model, left-horizontal two-threshold model, and two-threshold model are 4, 5, 6, and 7, respectively [33,77].

The threshold models with ΔAICc ≤ 2 confirmed the existence of SCES or thresholds. The threshold models with ΔAICc ≤ 7 were considered to have possible support for the existence of SCEs or thresholds [76,78].

Five groups (total bryophytes, total mosses, liverworts, acrocarpous mosses, and pleurocarpous mosses) were included in the analyses to explore the differences in the responses of SR to *choros* among different bryophyte groups.

The r^2^ and AICc values of the SKRs were compared with those of the corresponding SAR models to clarify whether the parameter *choros* is better than the area in predicting the SR of the bryophytes in the study region.

We fitted the above threshold models by using the function ‘sar_threshold’ of the R-package ‘sars’ [79] and visualized the models using the R package ‘ggplot2′ [80].

The semi-log model was used to detect the thresholds in the SKRs. *Choros* log transformation would possibly result in a bias influencing its prediction of SR [30]. To solve this deficiency, we used the null model suggested by Burns and colleagues [30]. The probability for a given island to possess a species depends on its relative *choros* value to the total *choros* value of all the studied islands (total number of habitat types * total area of the 66 islands) [30]. Thus, the island with a large *choros* attracts more species than the island with a small *choros*. The procedure was replicated 1000 times, and the average value of the number of species across all replicates was taken as the expected SR for each island. A deviation value was calculated for each island as follows [30]:

(1) $$DV_{i}=\frac{\left|OSR_{i}-ESR_{i}\right|}{C_{i}},$$

where *DV_i_*, *OSR_i_*, *ESR_i_,* and *C_i_* are the deviation value, observed SR, expected SR based on the null model, and the *choros* value for island *i* (*i* = 1, 2, 3, …*n*; *n* is the number of the islands with a focus group), respectively.

Then, the islands were divided into two island groups according to the first *choros* thresholds. Finally, ANOVA was used to test the differences in the deviation values between the island groups. If the deviation values of the island group below the *choros* threshold are significantly larger than those of the island group above the threshold, then the SCE is confirmed [30].

To understand the role of habitat per se in generating the SCEs in the SKRs, we used path analysis to detect the direct effects of habitat per se on SR after controlling the effect of island area on SR. The relationship of the SR with area and habitat could be expressed as follows:

Ln (*SR*) = *a* ln (*A*) + *b* ln (*H*), (2)

where *a* and *b* are the partial regression coefficients for area and habitat, respectively, and *A* and *H* are island area and the number of habitat types, respectively.

Based on the partial regression coefficients, the path coefficients of habitat (standardized partial regression coefficient) (P_habitat_) were calculated. Inspired by Triantis et al. and Chen [38,65], the procedure was performed by sequentially excluding islands from the largest *choros* to the smallest and meanwhile calculating the path coefficient of habitat (standardized partial regression coefficient of habitat). When P_habitat_ reaches a non-positive value, the corresponding *choros* is considered the upper limit of the SCE due to the direct effects of habitat. Similarly, we calculated the path coefficient of the area and tested whether there is a *choros* threshold of SCE due to the direct effects of area per se on SR.

## 5. Conclusions

This is the first study quantifying the relationships of SR with the combined effect of area and habitat (*choros*) by using piecewise regressions for the bryophytes on continental islands. We found the existence of two thresholds in the *choros* model for all five groups and the SCEs. The *choros* thresholds of the SCEs were higher in liverworts than in mosses and higher in pleurocarpous mosses than in acrocarpous mosses. *Choros* was essentially better than area or habitat alone as an explanatory variable in predicting the SR of the bryophytes in the study region. The detection of thresholds in SKRs was of practical value in the design of reserves, especially for bryophyte conservation.

## Figures and Tables

**Table 1:** Parameters of six SKR models for five bryophyte groups.

Models	Parameters	Total Bryophytes	Total Mosses	Liverworts	Acrocarpous Mosses	Pleurocarpous Mosses
Power model	R^2^_adj_	0.84	0.82	0.74	0.80	0.70
	AICc	503.93	496.06	147.70	450.75	355.90
	∆AICc	0	0.92	9.35	0	0
Simple logarithmic model	R^2^_adj_	0.71	0.73	0.36	0.73	0.54
	AICc	540.18	524.46	182.38	468.78	380.07
	∆AICc	36.25	29.32	44.03	18.03	24.17
Left-horizontal one-threshold model	*C*	12.45	10.82	2.38	8.52	3.57
	*T*_1_	1.47/4.34	1.17/3.21	5.54/254.17	1.07/2.91	1.67/5.30
	*Z*_2_	15.14	13.08	5.55	8.37	4.82
	R^2^_adj_	0.80	0.81	0.79	0.80	0.65
	AICc	518.68	504.60	142.47	454.08	367.82
	∆AICc	14.75	9.46	4.12	3.33	11.92
One-threshold model	*C*	12.18	11.58	1.71	8.95	3.54
	*T*_1_	1.59/4.91	1.48/4.40	5.63/278.11	1.41/4.09	5.63/278.11
	*Z*_1_	1.54	1.51	0.24	1.42	2.28
	*Z*_2_	15.03	13.45	5.43	8.50	12.31
	R^2^_adj_	0.80	0.81	0.80	0.79	0.69
	AICc	512.05	503.73	142.47	456.24	361.57
	∆AICc	8.12	8.59	4.12	5.49	5.67
Left-horizontal two-threshold model	*C*	12.29	11.31	1.880	8.28	3.82
	*T*_1_	0.97/2.63	0.87/2.38	2.44/11.45	0.67/1.95	1.17/3.21
	*T*_2_	5.77/319.58	5.87/353.19	5.64/280.90	6.07/431.39	5.67/289.17
	*Z*_2_	11.37	10.63	0.610	6.97	3.29
	*Z*_3_	32.57	27.84	5.090	16.95	11.41
	R^2^_adj_	0.85	0.84	0.830	0.820	0.72
	AICc	504.04	495.14	139.57	451.03	359.40
	∆AICc	0.11	0	1.22	0.28	3.5
Two-threshold model	*C*	12.17	11.59	2.872	8.742	3.83
	*T*_1_	1.03/2.79	1.02/2.77	2.024/7.57	0.91/2.49	1.26/3.52
	*T*_2_	5.77/320.86	5.87/353.19	5.63/278.11	6.07/432.25	5.63/278.38
	*Z*_1_	1.54	1.44	−0.73	1.04	0.35
	*Z*_2_	11.09	10.59	0.70	7.07	3.24
	*Z*_3_	32.87	27.88	5.03	16.83	11.31
	R^2^_adj_	0.85	0.84	0.84	0.81	0.70
	AICc	506.09	497.38	138.35	453.32	361.85
	ΔAICc	2.16	2.24	0	2.57	5.95

Note: Left-horizontal one-threshold model, *S* = *C* + | *Z*_2_·(ln*K*–*T*_1_) | (ln*K* > *T*_1_). Ordinary one-threshold model, *S* = *C* + *Z*_1_·ln*K* (ln*K* ≤ *T*_1_) + | *Z*_1_·*T*_1_ + *Z*_2_·(ln*K–T*_1_) | (ln*K* > *T*_1_).Left-horizontal two-threshold model, *S* = *C* + | *Z*_2_·(ln*K*–*T*_1_) | (*T_2_* ≥ ln*K* > *T*_1_) + | *Z*_2_·*T*_2_ + *Z*_3_·(ln*K*–*T*_2_) | (ln*K* > *T*_2_).Ordinary two-threshold model, *S* = *C* + *Z*_1_·ln*K* (ln*K* ≤ *T*_1_) + | *Z*_1_·*T*_1_ + *Z*_2_·(ln*K*-*T*_1_) | (*T*_2_ ≥ ln*K* > *T*_1_) + | *Z*_2_·*T*_2_ + *Z*_3_·(ln*K*–*T*_2_) | (ln*K* > *T*_2_). *S* is species richness, *K* is choros (number of habitat types * island area, nh*km^2^), *C* is an intercept, *T*_1_ and *T*_2_ are the first and second breakpoints, respectively, and *Z*_1_, *Z*_2_, and *Z*_3_ are slopes of the first, second, and third segments, respectively.

**Table 2:** The differences of the adjusted r^2^ values in SKRs minus those in SARs.

Model Types	Groups					Average
	Total Bryophytes	Total Mosses	LiverWorts	Acrocarpous Mosses	Pleurocarpous Mosses	
Power model	0.03	0.03	−0.01	0.04	0.02	0.022
Logarithmic model	0.01	0.02	−0.02	0.02	−0.01	0.004
Left-horizontal one-threshold model	0.02	0.02	0	0.03	0.01	0.016
One-threshold model	−0.011	0.025	0.003	0.028	0.01	0.011
Left-horizontal two-threshold model	0.02	0.02	0.01	0.03	0.02	0.020
Two-threshold model	0.026	0.03	0.008	0.032	0.016	0.0224

**Table 3:** The differences of the AICc values in SKRs minus those in SARs.

Model Types	Groups					Average
	Total Bryophytes	Total Mosses	Liverworts	Acrocarpous Mosses	Pleurocarpous Mosses	
Power model	−9.6	−10.43	1.00	−9.94	−2.46	−6.286
Logarithmic model	−2.33	−3.52	1.17	−4.65	0.76	−1.714
Left-horizontal one-threshold model	−6.4	−7.81	−0.38	−8.03	−1.27	−4.778
One-threshold model	−7.57	−8.07	−0.65	−8.15	−1.87	−5.262
Left-horizontal two-threshold model	−10.2	−11.1	−1.3	−9.92	−3.01	−7.106
Two-threshold model	−10.35	−11.11	−1.82	−9.96	−3.04	−7.256

## Data Availability

The data presented in this study are available from the corresponding author upon request.

## Supplementary Materials

The following supporting information can be downloaded at: https://www.mdpi.com/article/10.3390/plants12040837/s1. Figure S1: Six SAR models for five categories of bryophytes on 66 islands; Figure S2: Six SHR models for five categories of bryophytes on 66 islands; Figure S3: Path coefficient of habitat for five bryophyte categories; Figure S4: Path coefficient of area for five bryophyte categories; Table S1: Environmental information on 66 islands in central and southern Zhejiang; Table S2: Description of the 25 habitat types; Table S3: Relationships of accumulative species number with accumulative specimens; Table S4: Distribution of the 25 habitat types on 66 islands; Table S5: Occurrences of 236 bryophyte species on 66 islands in central and southern Zhejiang; Table S6: Species richness of the five bryophyte categories; Table S7: Difference of deviation values between small and large *choros* island groups; Table S8: Parameters of six SAR models for five bryophyte categories; Table S9: Parameters of six SHR models for five bryophyte categories; Table S10: The differences of the AICc values in SKRs minus those in respective SHRs; Table S11: The differences of the adjusted r^2^ values in SKRs minus those in respective SHRs; Table S12: The serial number and corresonding choros values of the islands with different categories of the bryophytes.
