# Supplementary material for: Thresholds in the Species–Area–Habitat Model: Evidence from the Bryophytes on Continental Islands
Source: Plants (Basel). 2023 Feb 13;12(4):837. doi: 10.3390/plants12040837 (PMC9962199; doi:10.3390/plants12040837)
Supplement: Supplementary file 1 [file plants-12-00837-s001.zip › Table S1 Environmental variables in 66 islands in the central and southern Zhejiang.pdf]

Table S1 Environmental variables in 66 islands in the central and southern Zhejiang

no	Islands	Lontidue (º)	Latitude (º)	Area (Km^2)	Number of residents	Number of Habitat types	Choros values ($nh.km^2$)
1	Xiajutou	121.59848	28.551283	0.02	-	8	0.16
2	Poyu	121.03501	27.437703	0.03	-	6	0.18
3	Beidouyu	121.69439	28.273475	0.03	-	7	0.21
4	Banbianyu	121.86091	28.704478	0.08	-	4	0.32
5	Dawuyu	121.64024	28.254156	0.05	-	9	0.45
6	Yisuandao	121.66229	28.214489	0.09	-	7	0.63
7	Xiamaanyu	121.01346	27.420844	0.14	-	5	0.7
8	Gaoshadao	121.66029	28.303125	0.07	-	12	0.84
9	Pingyu	121.06654	27.422869	0.11	-	8	0.88
10	Dongjishanxiaodao	121.91756	28.705767	0.09	-	10	0.90
11	Luoyu	121.73228	28.270606	0.17	-	6	1.02
12	Xiaodongjidao	121.91144	28.699986	0.16	-	7	1.12
13	Zhongqudao	121.08964	27.803058	0.113	-	10	1.13
14	Qianmenshandao	121.08857	27.871617	0.1	-	13	1.30
15	Jianyu	121.05357	27.426703	0.08	-	17	1.36
16	Shenmenshandao	121.09576	27.872614	0.11	-	13	1.43
17	Xiaoqudao	121.09154	27.808947	0.16	-	9	1.44
18	Hudongyu	121.14231	27.773808	0.224	-	8	1.792
19	Waidiaobangdao	121.69196	28.269389	0.23	-	9	2.07
20	Yumendao	121.24702	28.056862	0.15	-	14	2.10
21	Maohoushan	121.88808	28.512839	0.31	-	9	2.79
22	Ersuandao	121.64694	28.222419	0.27	-	13	3.51
23	Sanshantoudao	121.59766	28.562199	0.32	-	11	3.52
24	Menyu	121.06322	27.443439	0.32	-	11	3.52
25	Shitandao	121.81848	28.763528	0.55	-	8	4.40
26	Latoushandao	121.65678	28.293903	0.39	-	17	6.63
27	Gehaishandao	121.64666	28.299383	0.46	800	16	7.36
28	Zhongaodao	121.62829	28.461425	0.63	634	12	7.56
29	Xiaoludao	121.40227	28.097028	0.57	-	14	7.98
30	Nanshahuodao	121.68159	28.376497	0.51	513	16	8.16
31	Chaiyu	121.07835	27.43192	0.65	-	13	8.45
32	Niushandao	121.67923	28.283511	1.08	-	8	8.64
33	Yangyu	121.39282	28.127004	0.62	-	14	8.68
34	Zhidashandao	121.64816	28.359161	0.63	-	14	8.82
35	Beicedao	121.13376	27.774372	0.835	-	11	9.185
36	Changyu	121.88585	28.757364	1.17	-	9	10.53
37	Hengmencundao	121.66191	28.410342	0.61	1234	18	10.98
38	Sansuandao	121.63431	28.230611	0.81	143	14	11.34
39	Jishanxiang	121.35668	28.098203	1.66	7371	7	11.62
40	Yijiangshandao	121.80926	28.607844	0.91	-	16	14.56
41	Nancedao	121.13256	27.762542	1.04	575	15	15.60
42	Daludao	121.40428	28.087364	1.01	3000	18	18.18
43	Nangangshan	121.65979	28.422397	1.04	651	18	18.72
44	Tongmendao	121.67584	28.412608	1.05	528	19	19.95
45	Beigangdao	121.65119	28.431508	1.11	1200	18	19.98
46	Dongjidao	121.92306	28.716581	1.92	42	15	28.80
47	Zhangshutou	121.60868	28.496297	2.33	1311	15	34.95
48	Baiguoshandao	121.62396	28.485811	2.21	1400	17	37.57
49	Huangjiaodao	121.65171	28.469233	2.13	200	18	38.34
50	Sanpandao	121.15746	27.869592	1.86	1097	22	40.92
51	Daqudao	121.08304	27.786075	2.33	588	18	41.94
52	Pishandao	121.50764	28.091311	2.44	-	18	43.92
53	Banpingdao	121.13787	27.800933	2.44	4801	22	53.68
54	Queeraodao	121.85319	28.794447	4.55	242	14	63.70
55	Tianaodao	121.85699	28.739478	4.45	310	17	75.65
56	Toumendao	121.78522	28.688889	5.142	242	18	92.556
57	Xiadachengdao	121.89548	28.446017	4.895	2400	20	97.90
58	Xiaomendao	121.06497	27.996319	5.62	800	22	123.64
59	Chaoyanhoushan	121.61571	28.520897	5.57	5769	23	128.11
60	Shangdachendao	121.89478	28.492939	7.12	1508	22	156.64
61	Nanjidao	121.07146	27.467258	7.64	2000	22	168.08
62	Yuanjuedao	121.12863	27.885661	10.196	5956	22	224.312
63	Niyudao	121.04611	27.860419	12.64	12169	22	278.08
64	Dongtoudao	121.14921	27.838694	29.9	108200	23	687.70
65	Damendao	121.09464	27.969672	28.77	25820	24	690.48
66	Yuhuandao	121.19964	28.114247	184.55	613000	25	4613.75

Note： nh = number of habitat types
