# Supplementary material for: Thresholds in the Species–Area–Habitat Model: Evidence from the Bryophytes on Continental Islands
Source: Plants (Basel). 2023 Feb 13;12(4):837. doi: 10.3390/plants12040837 (PMC9962199; doi:10.3390/plants12040837)
Supplement: Supplementary file 1 [file plants-12-00837-s001.zip › Table S10. The differences of the AICc values in SKRs minus those in SHRs.pdf]

Table S10. The differences of the AICc values in SKRs minus those in SHRs

Model types	Categories					Average
	Total bryophytes	Total mosses	Liverworts	Acrocarpous mosses	Pleurocarpous mosses	
Power model	-42.6	-33.84	-29.81	-25.15	-23.23	-30.926
Logarithmic model	-41.04	-41.42	-11.65	-39.3	-22.12	-31.106
Left-horizontal one-threshold model	-31.51	-28.23	0.04	-22.07	7.09	-14.936
One-threshold model	0.64	-25.89	-1.13	-5.74	15.32	-3.360
Left-horizontal two-threshold model	-5.88	-3.82	NA	-8.09	12.72	-1.268
Two-threshold model	-4.37	-2.07	NA	-6.28	14.57	0.463
