# Supplementary material for: Thresholds in the Species–Area–Habitat Model: Evidence from the Bryophytes on Continental Islands
Source: Plants (Basel). 2023 Feb 13;12(4):837. doi: 10.3390/plants12040837 (PMC9962199; doi:10.3390/plants12040837)
Supplement: Supplementary file 1 [file plants-12-00837-s001.zip › Table S11. The differences of the adjusted r2 values in SKRs minus those in SHRs.pdf]

Table S11. The differences of the adjusted r^2 values in SKRs minus those in SHRs

Model types	Categories					Average
	Total bryophytes	Total mosses	Liverworts	Acrocarpous mosses	Pleurocarpous mosses	
Power model	0.15	0.11	0.31	0.1	0.15	0.164
Logarithmic model	0.24	0.24	0.24	0.22	0.21	0.230
Left-horizontal one-threshold model	0.12	0.11	0	0.09	0.08	0.080
One-threshold model	-0.023	0.086	0.017	0.023	-0.073	0.006
Left-horizontal two-threshold model	0.01	0	0.83	0.02	-0.06	0.160
Two-threshold model	0.015	0.01	0.835	0.026	-0.058	0.166
