# Supplementary material for: Thresholds in the Species–Area–Habitat Model: Evidence from the Bryophytes on Continental Islands
Source: Plants (Basel). 2023 Feb 13;12(4):837. doi: 10.3390/plants12040837 (PMC9962199; doi:10.3390/plants12040837)
Supplement: Supplementary file 1 [file plants-12-00837-s001.zip › Table S3 Relationships of accumulative species number with accumulative specimens.pdf]

Table S3. Relationships of accumulative species number with accumulative sampling efforts for eight largest islands

Islands / km^2	Total collected species / total specimen number	Asymptotic model	Maximum expected total species number	**Error %**
Yuhuandao / 184.55	154 / 1018	Y = 156.853 – 140.283*exp(-exp(-5.826)*x)	156.85	1.82
Dongtoudao / 29.0	87/400	Y = 90.532 – 84.304*exp(-exp(-4.999)*x)	90.53	3.90
Damendao / 28.77	96 / 563	Y = 98.984 – 89.96*exp(-exp(-5.288)*x)	98.99	3.02
Yuanjueidao /10.196	48/154	Y = 52.420 – 52.266*exp(-exp(-4.304)*x)	52.42	8.43
Nanjidao / 7.64	75/465	Y = 77.353 – 68.586*exp(-exp(-5.160)*x)	77.35	3.04
Shangdachendao / 7.12	53/256	Y = 54.284-49.607 *exp(-exp(-4.519)*x)	54.28	2.36
Xiaomendao / 5.62	62/220	Y =66.373 – 62.802*exp(-exp(-4.554)*x)	66.37	6.58
Chaoyanhoushan / 5.57	94/282	Y = 104.606-102.593 *exp(-exp(-4.937)*x)	104.61	10.13

Error percentage = (E – O) / E * 100%, E: Maximum expected total species number, O: Total collected species.
