# Supplementary material for: Thresholds in the Species–Area–Habitat Model: Evidence from the Bryophytes on Continental Islands
Source: Plants (Basel). 2023 Feb 13;12(4):837. doi: 10.3390/plants12040837 (PMC9962199; doi:10.3390/plants12040837)
Supplement: Supplementary file 1 [file plants-12-00837-s001.zip › Table S6. Species richness of the five bryophyte categories in 66 islands.pdf]

Table S6. Species richness of the bryophytes in the central and southern islands of Zhejiang Province, China

island	Bryophytes	Mosses	Acorcarpous mosses	Pleurocarpous mosses	Liverworts
Queeraodao	33	30	13	17	3
Shitandao	11	11	10	1	0
Changyu	9	8	4	4	1
Tianaodao	55	52	37	15	3
Dongjidao	40	38	29	9	2
Dongjishanxiaodao	16	15	9	6	1
Banbianyu	10	10	7	3	0
Xiaodongjidao	13	13	7	6	0
Toumendao	37	35	27	8	2
Yijiangshandao	35	34	29	5	1
Sanshantoudao	16	16	15	1	0
Xiajutou	7	7	6	1	0
Chaoyanhoushan	93	88	49	39	5
Maohoushan	6	6	6	0	0
Zhangshutou	16	16	13	3	0
Shangdachendao	53	50	37	13	3
Baiguoshandao	47	42	32	10	5
Huangjiaodao	46	46	32	14	0
Zhongaodao	27	26	23	3	1
Xiadachengdao	58	56	44	12	2
Beigangdao	36	36	31	5	0
Nangangshan	47	46	33	13	1
Tongmendao	35	35	25	10	0
Hengmencundao	30	29	20	9	1
Nanshahuodao	27	26	21	5	1
Zhidashandao	31	27	16	11	4
Gaoshadao	11	11	11	0	0
Gehaishandao	31	30	22	8	1
Latoushandao	25	25	20	5	0
Niushandao	10	8	8	0	2
Beidouyu	12	12	8	4	0
Luoyu	8	8	7	1	0
Waidiaobangdao	15	12	11	1	3
Dawuyu	4	4	4	0	0
Sansuandao	41	40	33	7	1
Ersuandao	16	16	13	3	0
Yisuandao	28	25	22	3	3
Yangyu	24	24	17	7	0
Yuhuandao	154	136	86	50	18
Jishanxiang	49	48	36	12	1
Xiaoludao	23	22	7	15	1
Pishandao	54	49	31	18	5
Daludao	43	40	23	17	3
Yumendao	22	19	11	8	3
Xiaomendao	62	60	44	16	2
Damendao	96	87	56	31	9
Yuanjuedao	47	47	35	12	0
Shenmenshandao	13	13	12	1	0
Qianmenshandao	18	18	16	2	0
Sanpandao	37	37	31	6	0
Niyudao	41	39	30	9	2
Dongtoudao	87	79	52	27	8
Xiaoqudao	19	16	7	9	3
Zhongqudao	19	15	9	6	4
Banpingdao	50	49	33	16	1
Daqudao	55	51	33	18	4
Beicedao	23	22	16	6	1
Hudongyu	4	4	4	0	0
Nancedao	29	29	18	11	0
Nanjidao	75	69	50	19	6
Menyu	16	16	11	5	0
Poyu	10	10	6	4	0
Chaiyu	21	20	15	5	1
Jianyu	3	3	3	0	0
Pingyu	7	7	7	0	0
Xiamaanyu	3	3	3	0	0
