# Supplementary material for: Thresholds in the Species–Area–Habitat Model: Evidence from the Bryophytes on Continental Islands
Source: Plants (Basel). 2023 Feb 13;12(4):837. doi: 10.3390/plants12040837 (PMC9962199; doi:10.3390/plants12040837)
Supplement: Supplementary file 1 [file plants-12-00837-s001.zip › Table S7. Difference of deviation values between small and large choros island groups.pdf]

Table S7. Deviation values in small and large choros island groups.

	small choros island group	large choros island group
Total bryophytes	18.11 ± 3.80^{A}	1.96 ± 0.21^{B}
Total mosses	18.19 ± 3.87^{A}	1.91 ± 0.21^{B}
Liverworts	0.53 ± 0.22^{a}	0.07 ± 0.01^{b}
Acrocarpous mosses	$13.57 \pm 2.75^{`A}$	1.44 ± 0.17^{B}
Pleurocarpous mosses	3.97 ± 1.19^{A}	0.46 ± 0.06^{B}

Note:

Data are the means of the values (|observed SR-expected SR |/ choros)

Data are presented as the mean ± standard error.

Different lowercase letters indicate statistical differences at 0.1 level ($p < 0.1$).

Different capital letters indicate statistical differences at 0.001 level ($p < 0.001$).
