# Supplementary material for: Thresholds in the Species–Area–Habitat Model: Evidence from the Bryophytes on Continental Islands
Source: Plants (Basel). 2023 Feb 13;12(4):837. doi: 10.3390/plants12040837 (PMC9962199; doi:10.3390/plants12040837)
Supplement: Supplementary file 1 [file plants-12-00837-s001.zip › Table S8. Parameters of threshold SAR models for five bryophyte categories.pdf]

Table S8 Parameters of threshold SAR models for five bryophyte categories

Models	Parameters	Total bryophytes	Total mosses	Liverworts	Acrocarpous mosses	Pleurocarpous mosses
Power model	R^2_{adj}	0.81	0.79	0.75	0.76	0.68
	AICc	513.53	506.49	146.70	460.69	358.36
	ΔAICc	0	0.25	6.53	0	0
Simple logarithmic model	R^2_{adj}	0.70	0.71	0.38	0.71	0.55
	AICc	542.51	527.98	181.21	473.43	379.31
	ΔAICc	28.98	21.74	41.04	12.74	20.95
Left-horizontal one-threshold model	C	12.300	11.63	2.370	8.590	3.860
	T_1	-1.112 / 0.328	-1.212 / 0.298	2.392 / 10.935	-1.412 / 0.244	-0.812 / 0.444
	Z_2	16.99	15.19	5.640	9.490	5.580
	R^2_{adj}	0.780	0.79	0.790	0.770	0.640
	AICc	525.08	512.41	142.85	462.11	369.09
	ΔAICc	11.55	6.17	2.68	1.42	10.73
One-threshold model	C	33.74	13.967	2.359	10.076	9.602
	T_1	2.588 / 13.303	-1.171 / 0.310	2.537 / 12.642	-1.363 / 0.256	2.537 / 12.642
	Z_1	9.680	1.132	0.282	0.662	2.715
	Z_2	36.96	15.050	5.709	9.445	12.879
	R^2_{adj}	0.810	0.780	0.795	0.761	0.677
	AICc	519.62	511.80	143.12	464.39	363.44
	ΔAICc	6.09	5.56	2.95	3.7	5.08
Left-horizontal two-threshold model	C	12.36	11.79	1.890	8.59	3.93
	T_1	-1.512 / 0.220	-1.512 / 0.220	-0.108 / 0.898	-1.712 / 0.181	-1.312 / 0.269
	T_2	2.588 / 13.303	2.688 / 14.702	2.592 / 13.356	2.888 / 17.957	2.588 / 13.303
	Z_2	12.83	12.24	0.75	7.99	3.77
	Z_3	33.79	28.74	5.44	17.53	12.10
	R^2adj	0.830	0.820	0.82	0.790	0.70
	AICc	514.24	506.24	140.87	460.95	362.41
	ΔAICc	0.71	0	0.7	0.26	4.05
Two-threshold model	C	16.607	14.977	1.167	11.399	5.203
	T_1	-1.436 / 0.238	-1.496 / 0.224	-0.337 / 0.714	-1.534 / 0.216	-1.182 / 0.307
	T_2	2.579 / 13.184	2.651 / 14.168	2.537 / 12.642	2.872 / 17.672	2.537 / 12.642
	Z_1	1.893	1.492	-0.739	1.124	0.572
	Z_2	12.597	11.924	0.891	8.071	3.711
	Z_3	33.892	28.712	5.251	17.358	11.948
	R^2_{adj}	0.819	0.807	0.827	0.775	0.685
	AICc	516.44	508.49	140.17	463.28	364.89
	ΔAICc	2.91	2.25	0	2.59	6.53

Note:

Power model: $S = C \cdot A^{\wedge Z}$

Simple logarithmic model: $S = C + Z_1 \cdot \ln A$

Left-horizontal one-threshold model: $S = C + | Z_2 \cdot (\ln A - T_1) | (\ln A > T_1)\ (Z_1 = 0)$

One-threshold model: $S = C + Z_1 \cdot \ln A\ (\ln A \leq T_1) + |Z_1 \cdot T + Z_2 \cdot (\ln A - T_1)| (\ln A > T_1)$

Left-horizontal two-threshold model: $S = C + | Z_2 \cdot (\ln A - T_1) | (T_2 \geq \ln A > T_1) + |Z_2 \cdot T_2 + Z_3 \cdot (\ln A - T_2) | (\ln A > T_2)$

Two-threshold model: $S = C + Z_1 \cdot \ln A\ (\ln A \leq T_1) + |Z_1 \cdot T_1 + Z_2 \cdot (\ln A - T_1) | (T_2 \geq \ln A > T_1) + |Z_2 \cdot T_2 + Z_3 \cdot (\ln A - T_2) | (\ln A > T_2)$

Where S is species richness, A is island area (km^2), C is an intercept; T_1 and T_2 are the first and second breakpoints, respectively; Z_1, Z_2 and Z_3 are slopes of the first, second and third segments, respectively. The data for T_1 and T_2 are ln (area, km^2) / area (km^2).

Parameters	Total bryophytes	Total mosses	Liverworts	Acrocarpous mosses	Pleurocarpous mosses
R^2_{adj}	0.81	0.79	0.75	0.76	0.68
AICc	513.53	506.49	146.70	460.69	358.36
R^2_{adj}	0.70	0.71	0.38	0.71	0.55
AICc	542.51	527.98	181.21	473.43	379.31
R^2_{adj}	0.780	0.79	0.790	0.770	0.640
AICc	525.08	512.41	142.85	462.11	369.09
R^2_{adj}	0.810	0.780	0.795	0.761	0.677
AICc	519.62	511.80	143.12	464.39	363.44
R^2adj	0.830	0.820	0.82	0.790	0.70
AICc	514.24	506.24	140.87	460.95	362.41
R^2_{adj}	0.819	0.807	0.827	0.775	0.685
AICc	516.44	508.49	140.17	463.28	364.89
