# Supplementary material for: Thresholds in the Species–Area–Habitat Model: Evidence from the Bryophytes on Continental Islands
Source: Plants (Basel). 2023 Feb 13;12(4):837. doi: 10.3390/plants12040837 (PMC9962199; doi:10.3390/plants12040837)
Supplement: Supplementary file 1 [file plants-12-00837-s001.zip › Table S9. Parameters of threshold SHR models for five bryophyte groupings.pdf]

Table S9. Parameters of six SHR models for five bryophyte categories.

Equation	Parameters	Total bryophytes	Total mosses	Liverworts	Acrocarpous mosses	Pleurocarpous mosses
Power model	R^2_{adj}	0.69	0.71	0.43	0.70	0.55
	AICc	546.53	529.90	177.51	475.90	379.13
	ΔAICc	36.61	30.94	35.08	16.78	32.88
Simple logarithmic model	R^2_{adj}	0.47	0.49	0.12	0.51	0.33
	AICc	581.22	565.88	194.03	508.08	402.19
	ΔAICc	71.3	66.92	51.6	48.96	55.94
Left-horizontal one-threshold model	C	14.518	13.332	2.294	10.274	7.385
	T	13.114	12.736	22.336	12.673	21.451
	Z2	6.157	5.517	5.451	3.619	12.013
	R^2_{adj}	0.680	0.700	0.790	0.710	0.570
	AICc	550.19	532.83	142.43	476.15	360.73
	ΔAICc	40.27	33.87	0	17.03	14.48
One-threshold model	C	-7.654	-1.993	1.421	-4.548	-1.583
	T	21.862	17.280	22.416	21.722	21.955
	Z1	2.554	1.864	0.058	1.712	0.685
	Z2	31.112	7.662	5.455	14.310	12.000
	R^2_{adj}	0.822	0.719	0.781	0.766	0.760
	AICc	511.41	529.62	143.60	461.98	346.25
	ΔAICc	1.49	30.66	1.17	2.86	0
Left-horizontal two-threshold model	C	12.970	11.831		9.070	4.360
	T_1	10.200	9.901		10.201	11.000
	T_2	22.000	22.000		22.402	22.000
	Z_2	3.450	3.270		2.400	0.940
	Z_3	30.540	25.951		16.600	11.660
	R^2_{adj}	0.840	0.840		0.800	0.780
	AICc	509.92	498.96		459.12	346.68
	ΔAICc	0	0		0	0.43
Two-threshold model	C	10.288	14.611		11.801	3.087
	T1	10.576	9.730		9.788	11.241
	T2	22.000	22.000		22.402	22.000
	Z1	0.360	-0.361		-0.369	0.146
	Z2	3.474	3.290		2.389	0.924
	Z3	30.510	25.915		16.636	11.691
	R^2_{adj}	0.830	0.827		0.781	0.759
	AICc	510.46	499.45		459.60	347.28
	ΔAICc	0.54	0.49		0.48	1.03

Note:

Left-horizontal one-threshold model: $S = C + | Z_2 \cdot (H - T_1) | (H > T_1)$

Ordinary one-threshold model: $S = C + Z_1 \cdot H\ (H \leq T_1) + | Z_1 \cdot T_1 + Z_2 \cdot (H - T_1) | (H > T_1)$

Left-horizontal two-threshold model: $S = C + | Z_2 \cdot (H - T_1) | (T_2 \geq H > T_1) + | Z_2 \cdot T_2 + Z_3 \cdot (H - T_2) | (H > T_2)$

Ordinary two-threshold model: $S = C + Z_1 \cdot H\ (H \leq T_1) + |Z_1 \cdot T_1 + Z_2 \cdot (H-T_1)| (T_2 \geq H > T_1) + |Z_2 \cdot T_2 + Z_3 \cdot (H - T_2)| (H > T_2)$

Where S is species richness, H is the number of habitat types, C is an intercept; T_1 and T_2 are the first and second breakpoints, respectively; Z_1, Z_2 and Z_3 are slopes of the first, second and third segments, respectively.
