# Supplementary figures and images for: Thresholds in the Species–Area–Habitat Model: Evidence from the Bryophytes on Continental Islands
Source: Plants (Basel). 2023 Feb 13;12(4):837. doi: 10.3390/plants12040837 (PMC9962199; doi:10.3390/plants12040837)

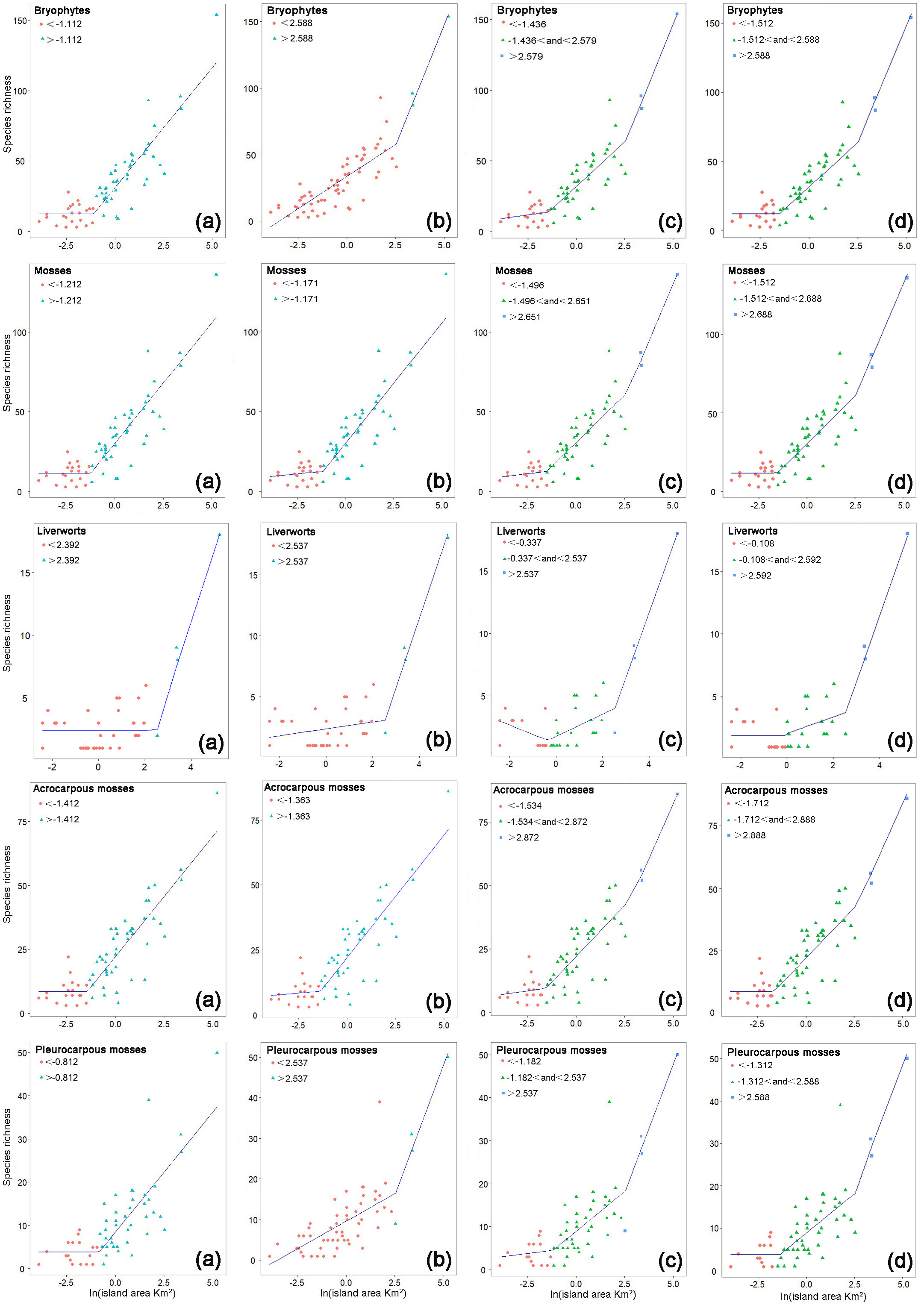

Supplement: Supplementary file 1 [file plants-12-00837-s001.zip › Figure S1. Threshold SAR models for the five bryophyte categories.jpg]

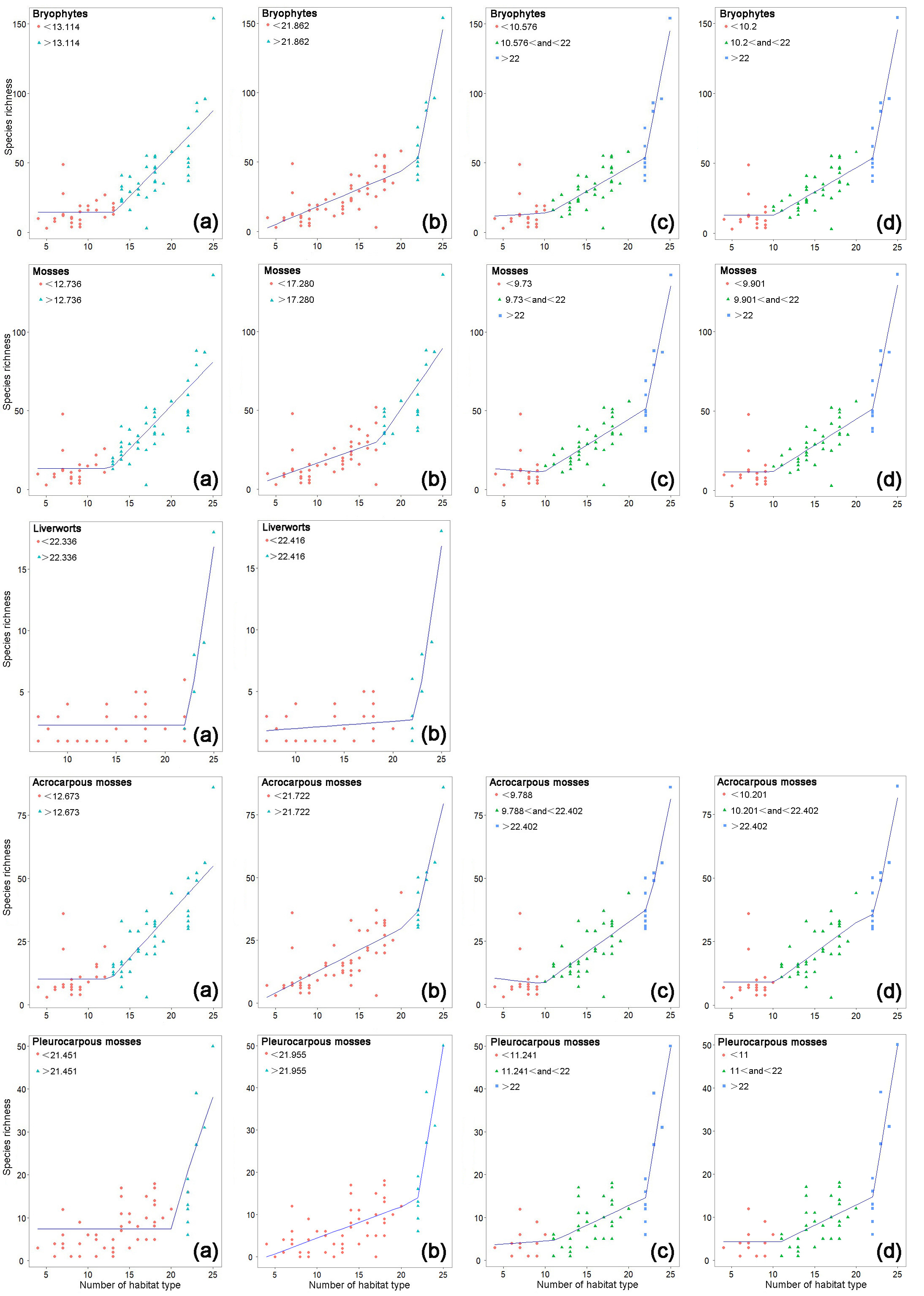

Supplement: Supplementary file 1 [file plants-12-00837-s001.zip › Figure S2. Threshold SHRs for five bryophyte categories.jpg]

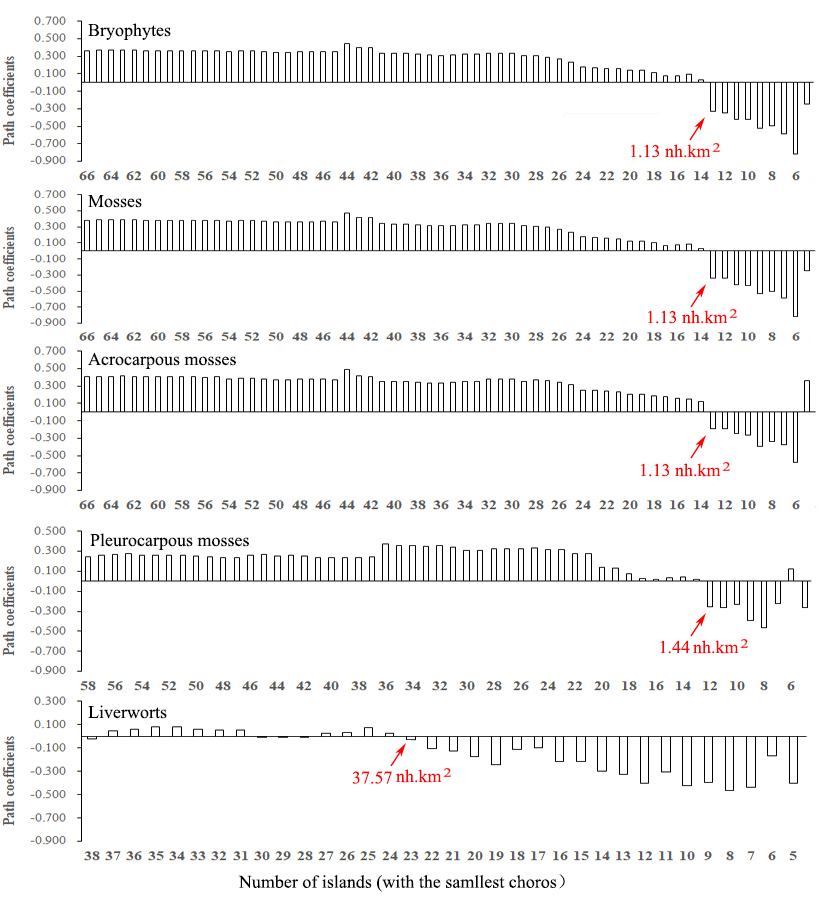

Supplement: Supplementary file 1 [file plants-12-00837-s001.zip › Figure S3. Path coefficients of habitat against area relevant to bryophyte SRs.jpg]

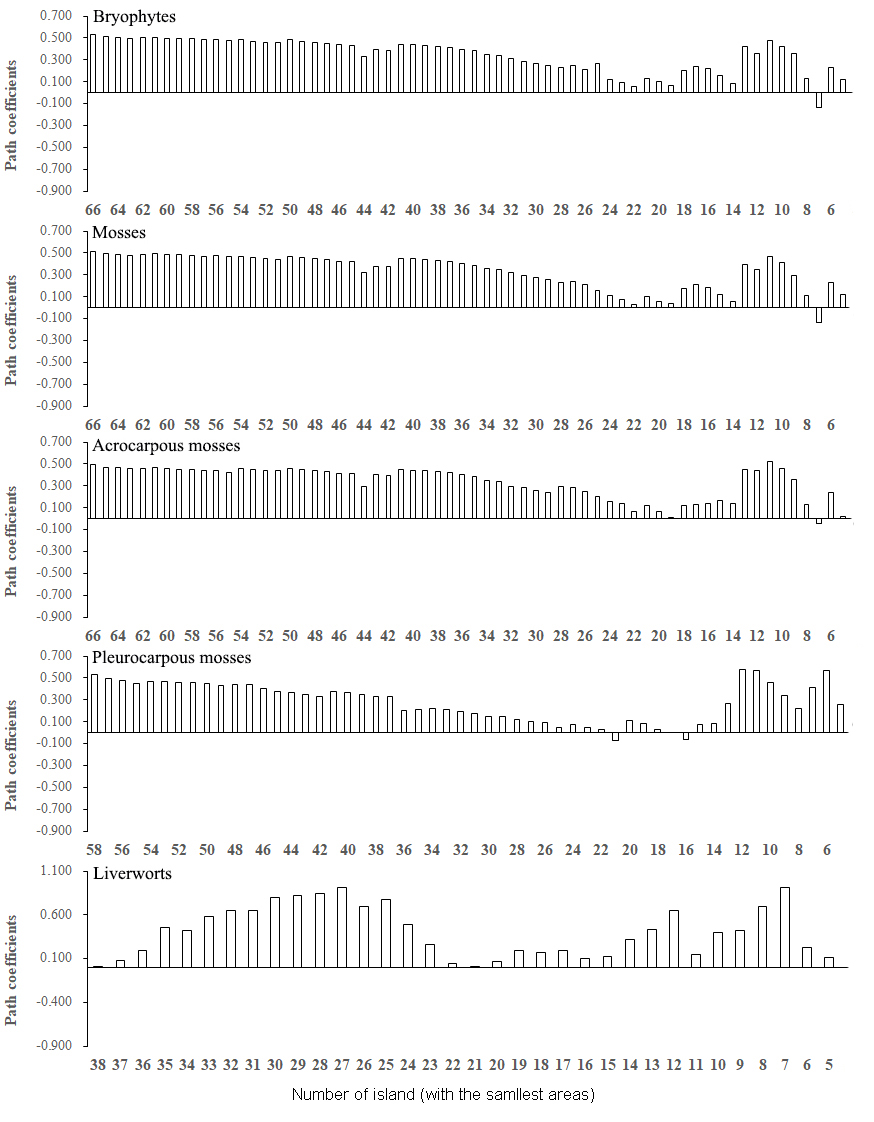

Supplement: Supplementary file 1 [file plants-12-00837-s001.zip › Figure S4. Path coefficients of area against habitat relevant to bryophyte SR s.jpg]
